# Performance characteristics of a polymerase chain reaction-based assay for the detection of *EGFR* mutations in plasma cell-free DNA from patients with non-small cell lung cancer using cell-free DNA collection tubes

**DOI:** 10.1371/journal.pone.0295987

**Published:** 2024-04-09

**Authors:** Theresa May, Michelle S. Clement, Harkanwal Halait, Alexander Kohlmann, Milena Kohlmann, Jason Lai, Nitta Lee, Xiaocheng Li-Sucholeiki, Peter Meldgaard, Snehal Joshi, Sidney Scudder, Neelima Shrestha, Boe Sorensen, Marilyn Kiral, Patrick O’Donnell

**Affiliations:** 1 Research and Development, Roche Molecular Systems, Inc., Pleasanton, CA, United States of America; 2 Department of Clinical Biochemistry, Aarhus University Hospital, Aarhus, Denmark; 3 Oncology R&D, Precision Medicine and Biosamples, AstraZeneca, Gaithersburg, MD, United States of America; 4 Oncology R&D, Precision Medicine and Biosamples, AstraZeneca, Boston, MA, United States of America; 5 Department of Oncology, Aarhus University Hospital, Aarhus, Denmark; National Institute of Cancer Research, TAIWAN

## Abstract

Survival rates in non-small cell lung cancer (NSCLC) are low. Detection of circulating tumor DNA in liquid biopsy (plasma) is increasingly used to identify targeted therapies for clinically actionable mutations, including *EGFR* mutations in NSCLC. The cobas^®^ EGFR Mutation Test v2 (cobas EGFR test) is FDA-approved for *EGFR* mutation detection in tissue or liquid biopsy from NSCLC. Standard K2EDTA tubes require plasma separation from blood within 4 to 8 hours; however, Roche Cell-Free DNA (cfDNA) Collection Tubes (Roche cfDNA tube) enable whole blood stability for up to 7 days prior to plasma separation. This analysis assessed performance of Roche cfDNA tubes with the cobas EGFR test for the detection of *EGFR* mutations in plasma from healthy donors or patients with NSCLC. Overall, test performance was equally robust with either blood collection tube, eg, regarding limit of detection, linearity, and reproducibility, making Roche cfDNA tubes suitable for routine clinical laboratory use in this setting. Importantly, the Roche cfDNA tubes provided more flexibility for specimen handling versus K2EDTA tubes, eg, in terms of tube mixing, plasma separation, and sample stability, and do not require processing of blood within 8 hours thereby increasing the reach of plasma biopsies in NSCLC.

## Introduction

Lung cancer remains a leading cause of cancer-related deaths globally, accounting for 2.2 million new cases and 1.8 million deaths per year [1]. Non-small cell lung cancer (NSCLC) represents 82% of all lung cancers, and is typically diagnosed at an advanced and/or metastatic stage. Despite advances in treatment, the estimated 5-year survival rate for patients with NSCLC remains at just 26% [2].

Recent advances in the biologic understanding of NSCLC, driven in part by the evolution of advanced diagnostics such as next-generation sequencing (NGS), have shown the importance of the molecular features associated with the disease. Specifically, subsets of NSCLC with specific genetic alterations have been identified, including those that harbor abnormalities in *EGFR*, *ALK*, *MET*, *HER2*, *ROS1*, *BRAF*, *RET*, *NTRK1*, *PIK3CA*, *KRAS*, and *MEK* [3]. As key molecular drivers of the disease, these features have led to the development of molecularly targeted therapeutic approaches in NSCLC [4–12]. For instance, the most common oncogenic molecular alteration in NSCLC is in *EGFR*; these alterations are found in 10% to 50% of patients with NSCLC depending upon demographics such as ethnicity, gender, smoking history, and histology [13]. Clinical trials of agents targeted to *EGFR* have led to significantly improved survival versus standard-of-care chemotherapy in the *EGFR* mutation-positive population of patients with NSCLC [4–7]. Consequently, there are targeted therapies available that are directed at variants in the *EGFR* gene in newly diagnosed patients with non-squamous advanced NSCLC that have been approved by the US Food and Drug Administration (FDA) [12, 14] and, from 2018, testing of this gene has been considered a minimum requirement per guidelines from the College of American Pathologists, the International Association for the Study of Lung Cancer, and the Association for Molecular Pathology [14].

Although molecular analysis of tissue samples is regarded as standard practice in NSCLC, there may be limitations with this approach relating to sampling location, amount of tissue available for sampling, and tumor heterogeneity [15]. *EGFR* mutations can be detected in plasma as circulating tumor DNA (ctDNA), a technique also known as liquid biopsy. Notably, the use of liquid biopsy is supported in clinical guidelines for advanced lung cancer, and the assays Guardant360^®^ CDx (Guardant, Palo Alto, CA) and FoundationOne^®^Liquid CDx (Foundation Medicine, Inc., Cambridge, MA, USA) have received FDA approval as companion diagnostics across a range of biomarkers, including for *EGFR*-mutated NSCLC [16–20]. The cobas^®^ EGFR Mutation Test v2 (cobas EGFR test [Roche Molecular Diagnostics GmbH, Mannheim, Germany]) is FDA-approved specifically for the detection of defined *EGFR* mutations in tissue or liquid biopsy (plasma) from patients with NSCLC [21].

When collected in standard di-potassium salt of ethylene diamine tetraacetic acid (K2EDTA) blood draw tubes, plasma must be separated from blood within 4 to 8 hours. The Roche Cell-Free DNA (cfDNA) Collection Tube (Roche cfDNA tube [Roche Molecular Systems, Inc., Branchburg, NJ, US) contains an anticoagulant (tri-potassium salt of ethylene diamine tetra acetic acid [K3EDTA]), a cell lysis inhibitor, and a cfDNA stabilizer that allows whole blood to be stable for up to 7 days prior to separation of the plasma. However, it has not been previously reported whether K3EDTA or the cell lysis inhibitor affect the performance of assays designed to detect mutations in ctDNA. The results presented here demonstrate the performance of Roche cfDNA tubes, including a comparison with standard K2EDTA tubes, for the detection of defined *EGFR* mutations in plasma derived from patients with NSCLC or from healthy donors using the cobas EGFR test.

## Materials and methods

### Specimens and samples

Between May 2018 through February 2020, plasma samples from healthy donors or patients with stage III or IV NSCLC were derived from whole blood and collected into Roche cfDNA tubes; plasma from patients with NSCLC was co-collected into standard K2EDTA tubes for comparison with Roche cfDNA tubes (8 mL per tube for both tube types). Samples were obtained from commercial vendors, who previously obtained ethical approval for all protocols from their local Institutional Review Board or other appropriate ethics committee. All samples were collected with informed consent. To supplement the samples from patients with *EGFR* mutation-positive NSCLC, surrogate samples were prepared using whole blood from healthy *EGFR* mutation-negative donors spiked with sheared cell-line DNA to approximately 1.5× the limit of detection (LoD) of the corresponding target sequence detected and reported by the cobas EGFR test. The predominant *EGFR* mutation for each group (eg, exon 19 deletion [Ex19Del], L858R, G719X, and exon 20 insertion [Ex20Ins]) reported by the cobas EGFR test was used for each surrogate sample (Table 1). Cell-line DNA was procured from a commercial vendor (Horizon Discovery, Cambridge, UK), sheared, and characterized, eg, by calibration of ctDNA with a known quantitation of target mutation sequences, prior to use. For the clinical LoD confirmation analysis, the concentration of *EGFR* mutations present in NSCLC samples was similarly estimated to allow formulation of the panel at approximately 1× LoD. The mutation status of the samples from patients with NSCLC was confirmed by a validated in-house NGS test using the Illumina MiSeq System (Illumina, Inc., San Diego, CA, US).

**Table 1:** The predominant *EGFR* mutation for each group reported by the cobas EGFR test.

Exon	*EGFR* mutation group	*EGFR* nucleic acid sequence	COSMIC ID^a^
Exon 18	G719X	2156G>A	6252
Exon 19	Ex19Del	ex19del 2235_2249del15	6223
Exon 20	S768I	2303G>T	6241
	T790M	2369C>T	6240
	Ex20Ins	2307_2308insGCCAGCGTG	12376
Exon 21	L858R	2573T>G	6224
	L861Q	2582T>A	6213

^a^Catalogue Of Somatic Mutations In Cancer (COSMIC), https://cancer.sanger.ac.uk/cosmic, last accessed March 14, 2023.

COSMIC ID, Catalogue Of Somatic Mutations In Cancer identification; Ex19Del, exon 19 deletion; Ex20Ins, exon 20 insertion.

### Detection of *EGFR* mutations by the cobas EGFR test

The cobas EGFR test is a real-time polymerase chain reaction (PCR) assay consisting of two major processes: (1) manual specimen preparation to obtain cfDNA from 2 mL of plasma samples or genomic DNA from formalin-fixed paraffin-embedded tumor tissue; and (2) PCR-based amplification and detection of target DNA using complementary primer pairs and probes labeled with fluorescent dyes. For plasma samples, the Semi-Quantitative Index (SQI) (available in the CE-IVD version [21]), a semi-quantitative measure of the amount of mutation-positive cfDNA in a sample that correlates with copies (cp)/mL, was used to measure the presence of *EGFR* mutations in plasma.

### Detection of *EGFR* mutations by targeted DNA amplicon sequencing

Plasma from patients with NSCLC and healthy donors was tested using a validated, targeted DNA amplicon sequencing NGS technology to confirm detected *EGFR* mutations. For each NSCLC and healthy donor sample collected in K2EDTA tubes, PCR-based DNA input was used to amplify regions in each exon, followed by dual-indexing PCR. The sample libraries were pooled and sequenced along with positive controls, using the 2× 150 base pair paired-end sequencing protocol on the Illumina MiSeq System.

### Interference study

Samples for an interference study utilized plasma from whole blood from healthy donors collected with one lot of Roche cfDNA tubes. Sheared *EGFR* mutation-positive cell-line DNA was diluted into four sets of the collected plasma at 2× LoD, along with one set containing no *EGFR* mutation-positive cell-line DNA. The plasma was then spiked with potential interfering substances and tested along with control conditions (no interfering substances) and assessed with the cobas EGFR test. The following sample types were evaluated: control (no potentially interfering substance [with and without diluents used for substances, eg, normal saline or H_2_O]); hemoglobin (2 g/L); triglycerides (33 g/L [37 mmol/L]); albumin (60 g/L); bilirubin (both conjugated and unconjugated; 0.2 g/L [342 mmol/L]); bilirubin (unconjugated; 0.2 g/L [342 mmol/L]); preservative solution (4× the intended amount).

The test concentrations for hemoglobin, triglycerides, albumin, and bilirubin were from the Clinical and Laboratory Standards Institute (CLSI) EP07-A2 guideline [22]. Potential interference was assessed based on observing the expected mutation calls.

### Statistical analysis

Roche cfDNA tube performance was evaluated against predefined acceptance criteria. LoD verification was defined as the proportion of correct results as a percentage of the total number of valid results. For clinical LoD verification and the reproducibility study, data were summarized by the percentage agreement by panel member with the associated 95% exact confidence intervals. Linearity was evaluated according to CLSI document EP06-A [23] and confirmed by comparing the differences in predicted SQI values between the best-fit second- or third-order polynomial regression, as determined by the higher root mean square error value, and the predicted values based on the first order polynomial regression. Root mean square error was defined as a measurement of the differences between values in a predictive model. The fit was considered linear if the differences were <1.0 SQI.

In the method comparison study, the positive, negative, and overall percentage agreement (OPA) between the *EGFR* mutation results from plasma collected in Roche cfDNA tubes and plasma collected in K2EDTA tubes were calculated, with 95% confidence intervals also reported. The specimen stability study compared the results for each sample at each time point with the corresponding Time 0 result. The last time point for a given storage condition at and before which the results agreed was determined to be the stability for the storage condition. The mixing study compared the observed results for each test condition with the expected results. If the results agreed, the condition passed. The centrifugation study compared the observed results for each test condition with the expected results. If the results agreed, the condition passed. The surrogate sample study used a hit rate comparison to a reference panel using *P*-values from the Fisher’s exact test for levels that had less than a 100% hit rate. The interfering substances study compared the observed results in the presence and absence of potentially interfering substances or material with the expected results. If the results agreed, the potentially interfering substance or material was determined to not interfere with the cobas EGFR test.

## Results

### Surrogate samples

A comparison was made using *EGFR* mutation-positive samples (Ex19Del, L858R, and T790M) diluted from approximately 2× LoD to 0.03× LoD, along with an *EGFR* mutation-negative control, and four panels: 1) sheared cell-line DNA diluted into healthy donor plasma; 2) sheared cell-line DNA diluted into *EGFR* mutation-negative NSCLC plasma; 3) sheared cell-line DNA diluted into healthy donor whole blood prior to plasma separation; and 4) *EGFR* mutation-positive plasma diluted in *EGFR* mutation-negative NSCLC patient plasma samples (reference panel). All mutations were confirmed using sequencing.

Twenty replicates from each panel were tested across two lots of the cobas EGFR test reagents. The hit rates for each panel level and mutation were compared with the reference panel (S1–S3 Tables); all levels had a 100% hit rate or a *P* value ≥0.05, except for two panel levels for Ex19Del (approximately 0.03× LoD and 0.13× LoD in Panels 2 and 3, respectively). Based on this analysis, the results indicate that the surrogate samples perform similarly to the *EGFR* mutation-positive NSCLC plasma reference (Condition 4).

### Mixing

This study evaluated the minimum number of inversions needed to mix whole blood with anticoagulant after collection in the Roche cfDNA tube, and aimed to determine whether increased exposure to the Roche cfDNA tube rubber stopper through multiple inversions would impact the performance of the cobas EGFR test.

One lot of Roche cfDNA tubes was utilized to collect whole blood from healthy donors. After collection, each tube was gently inverted a specified number of times by hand. Testing was performed using surrogate samples of sheared *EGFR* mutation-positive cell-line DNA diluted into the whole blood at 2× LoD and mixed according to the specified number of inversions in S4 Table. Cell-line DNA was not added to the *EGFR* mutation-negative samples.

The performance of the cobas EGFR test was not affected by any of the conditions. A minimum of four total inversions was needed to mix the blood collected in the Roche cfDNA tube, as fewer than four inversions resulted in increased hemolysis (S1 Fig). There was also no impact to the cobas EGFR test results for G719X, Ex19Del, S768I, T790M, Ex20Ins, L858R, and L861Q mutations after increased exposure (30 inversions) to the tube rubber stopper.

### Centrifugation

A guard band study was performed to determine whether deviations from the recommended centrifugation operating range of ≤1600 x g for 10 to 15 minutes influenced the performance of the cobas EGFR test.

Samples for this study utilized whole blood from healthy donors collected with one lot of Roche cfDNA tubes. Sheared *EGFR* mutation-positive cell-line DNA was diluted into four sets of the collected whole blood at 2× LoD, along with one set containing no *EGFR* mutation-positive cell-line DNA. The collected whole blood was then centrifuged at various times and speeds (S5 Table), and the resulting plasma assessed with the cobas EGFR test. All results gave the correct mutation call for all tested samples (Ex19Del, Ex20Ins, S768I, G719A, L861Q, T790M, L858R, and wildtype) for each centrifugation condition. The tested centrifugation conditions had no impact on the performance of the cobas EGFR test.

### LoD verification study

The LoD, previously established with K2EDTA plasma, for the cobas EGFR test was verified using plasma from Roche cfDNA tubes for exon 18 G719X mutations, Ex19Del mutations, exon 20 S768I, T790M, Ex20Ins mutations, and exon 21 L858R and L861Q mutations. Twenty replicates were tested per mutation using surrogate samples and one batch of cobas EGFR test reagents. The LoD for *EGFR* mutation detection in Roche cfDNA tubes’ plasma was verified as ≤100 cp/mL, as shown in Table 2.

**Table 2:** Verification of LoD for *EGFR* mutation detection.

*EGFR* mutation	Test level (cp/mL)	Valid results (*n*)	Correct results (*n*)	Hit rate (%)	95% CI
Ex19Del	75	20	20	100	83.2, 100
S768I	25	20	20	100	83.2, 100
L858R	100	20	20	100	83.2, 100
T790M	100	20	20	100	83.2, 100
L861Q	30	20	20	100	83.2, 100
G719X	100	20	20	100	83.2, 100
Ex20Ins	25	20	20	100	83.2, 100

CI, confidence interval; cp, copies; Ex19Del, exon 19 deletion; Ex20Ins, exon 20 insertion; LoD, limit of detection.

### Clinical LoD verification study

This analysis aimed to verify the sensitivity of the cobas EGFR test using samples from patients with NSCLC, collected in Roche cfDNA tubes. Two panels of *EGFR* mutation-positive NSCLC specimens for Ex19Del, L858R, T790M, and S768I were diluted to approximately the LoD for each mutation group in pooled NSCLC *EGFR* mutation-negative plasma collected in two Roche cfDNA tube lots. Three unique Ex19Del mutations were evaluated, the two most predominant plus one additional mutation. *EGFR* mutation-negative NSCLC samples were tested in plasma from both lots of Roche cfDNA tubes. All mutations were confirmed by NGS. Panels were tested on 2 non-consecutive days by two operators at each of three testing sites using one lot of cobas EGFR test reagents. Table 3 shows the mutations tested, the target concentrations, and the OPA for each mutation; all mutations had an OPA of ≥95.8%.

**Table 3:** *EGFR* mutations tested, target concentrations, and OPA for each *EGFR* mutation.

*EGFR* mutation	Concentration (cp/mL)	Valid tests (*n*)	Agreement (*n*)	OPA, % (95% CI)
Wildtype	–	48	48	100 (92.6, 100)
Ex19del 2235_2249del15	75	48	48	100 (92.6, 100)
Ex19del 2236_2250del15	75	48	48	100 (92.6, 100)
Ex19del 2236_2253dup TTCCCGTCGCTATCAAGG	75	48	48	100 (92.6, 100)
L858R	100	48	47	97.9 (88.9, 99.9)
T790M	100	48	47	97.9 (88.9, 99.9)
S768I	25	48	46	95.8 (85.7, 99.5)

CI, confidence interval; cp, copies; Ex19Del, exon 19 deletion; OPA, overall percentage agreement.

### Linearity verification with Roche cfDNA tube plasma

Linearity of the cobas EGFR test, established in K2EDTA plasma, was verified using plasma from Roche cfDNA tubes. Surrogate samples were used to test each mutation group using two replicates at 1.0 × 10^5^ cp/mL (except for G719X, where the highest level tested was 1.0 × 10^4^ cp/mL) and four replicates at the remaining levels using two lots of cobas EGFR test reagents (S6 Table).

**Fig 1:**
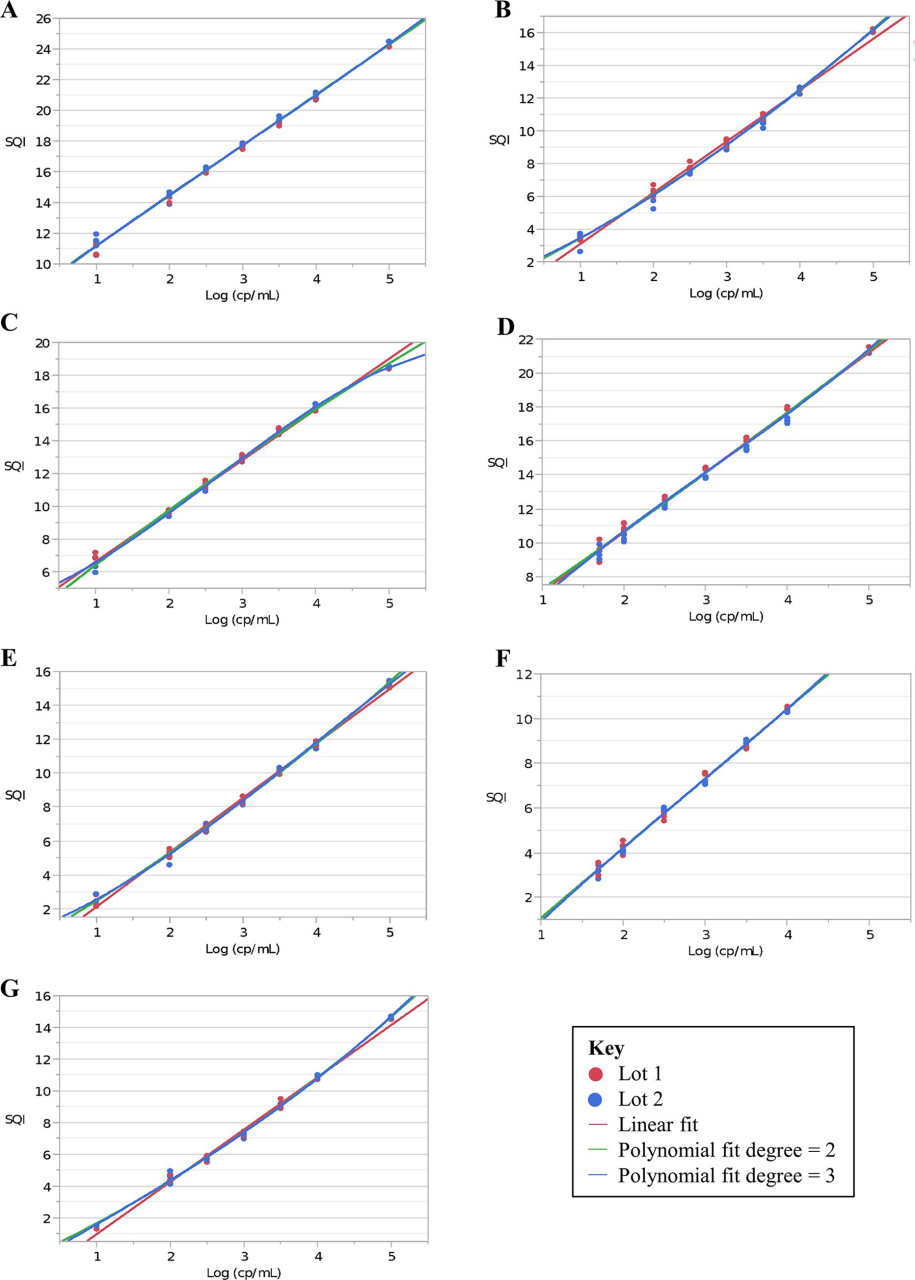
Linear fit and the best second- or third-order polynomial fit for the surrogate samples for *EGFR* mutations: A: Ex19Del; B: S768I; C: L858R; D: T790M; E: L861Q; F: G719X; and G: Ex20Ins. Ex19Del, exon 19 deletion; Ex20Ins, exon 20 insertion; SQI, Semi-Quantitative Index.

All mutations met the requirements, and the linearity for the ranges shown in S6 Table was verified. The linear fit and the best second- or third-order polynomial fit are shown in Fig 1, and the differences between the regression analyses are shown in S7–S13 Tables.

### Method comparison

The diagnostic accuracy of the cobas EGFR test using plasma from Roche cfDNA tubes was assessed by comparing results of *EGFR* mutation-positive NSCLC samples collected in Roche cfDNA tubes with samples collected in K2EDTA tubes simultaneously from the same donor.

A total of 17 patients with *EGFR* mutation-positive NSCLC and 34 patients with NSCLC who were mutation-negative for *EGFR* in plasma were included in this study. Only *EGFR*-positive mutations with at least one mutation that met a minimum requirement (S14 Table) to be consistently detectable at approximately 1× LoD were used in the analysis.

A total of 71 samples (51 from patients with NSCLC [mutation-positive NSCLC: 17 patients; *EGFR* mutation-negative in plasma: 34 patients] and 20 surrogate samples) were tested in plasma from both the Roche cfDNA and K2EDTA tubes. There was 100% agreement for all samples, as shown in Tables 4 and 5. Some samples contained more than one *EGFR* mutation, resulting in more than 71 total results. The agreement for each mutation is shown in Table 6.

**Table 4:** Agreement between the Roche cfDNA tubes and K2EDTA tubes.

		K2EDTA tubes		
		MD	NMD	Total
**Roche cfDNA tubes**	**MD**	37	0	37
	**NMD**	0	34	34
	**Total**	37	34	71

cfDNA, cell-free DNA; MD, mutation detected; NMD, no mutation detected.

**Table 5:** Overall agreement between all samples.

Agreement	Valid results (*n*)	Correct results (*n*)	Hit rate (%)	95% CI
Positive	37	37	100	90.5, 100
Negative	34	34	100	89.7, 100
Overall	71	71	100	94.9, 100

CI, confidence interval.

**Table 6:** Agreement between K2EDTA tubes and the Roche cfDNA tubes for each *EGFR* mutation.

		K2EDTA tubes								
		Ex19Del	S768I	L858R	T790M	L861Q	G719X	Ex20Ins	Wildtype	Total
**Roche cfDNA tubes**	**Ex19Del**	15	–	–	–	–	–	–	–	15
	**S768I**	–	2	–	–	–	–	–	–	2
	**L858R**	–	–	16	–	–	–	–	–	16
	**T790M**	–	–	–	16	–	–	–	–	16
	**L861Q**	–	–	–	–	2	–	–	–	2
	**G719X**	–	–	–	–	–	2	–	–	2
	**Ex20Ins**	–	–	–	–	–	–	2	–	2
	**Wildtype**	–	–	–	–	–	–	–	34	34
	**Total**	15	2	16	16	2	2	2	34	89

Dashed lines indicate that samples were not positive for the mutation detected.

cfDNA, cell-free DNA; Ex19Del, exon 19 deletion; Ex20Ins, exon 20 insertion.

### Reproducibility

Reproducibility of the Roche cfDNA tubes with the cobas EGFR test was evaluated with three lots of tubes, three testing sites, two operators per site, and on 3 non-consecutive days. One lot of the cobas EGFR test reagents was used. Three 9-member panels were made using surrogate samples of sheared *EGFR* mutation-positive cell-line DNA diluted into healthy donor plasma collected in three unique Roche cfDNA tube lots. Each mutation group was diluted to approximately 100 cp/mL and 300 cp/mL. Samples included mutation-negative, an exon 18 mutation (G719A), an Ex19Del, three exon 20 mutations (S768I, T790M, and Ex20Ins), and two exon 21 mutations (L858R and L861Q), some of which were combined to make individual panel members. Each panel was confirmed by sequencing.

The OPA for each mutation member is shown in Table 7. All panel members had a percentage agreement of ≥98.6%. The overall coefficient of variation for the cycle threshold from valid mutation members ranged from 4.4% to 13.7% across all mutation members (S15 Table). Within each component, coefficient of variation ranged from 0.0% to 10.4% across all mutation members.

**Table 7:** OPA for each mutation member.

Panel member–level	Valid tests (*n*)	Agreement (*n*)	OPA, % (95% CI)
Wildtype–N/A	72	71	98.6 (92.5, 100)
G719A – 100 cp/mL	72	71	98.6 (92.5, 100)
Ex19Del– 100 cp/mL	72	72	100 (95.0, 100)
Ex20Ins– 100 cp/mL	72	72	100 (95.0, 100)
S768I – 100 cp/mL	72	72	100 (95.0, 100)
T790M – 100 cp/mL	72	72	100 (95.0, 100)
L858R – 100 cp/mL	72	72	100 (95.0, 100)
L861Q – 100 cp/mL	72	72	100 (95.0, 100)
G719A – 300 cp/mL	72	72	100 (95.0, 100)
Ex19Del– 300 cp/mL	71	71	100 (94.9, 100)
Ex20Ins– 300 cp/mL	71	71	100 (94.9, 100)
S768I – 300 cp/mL	72	72	100 (95.0, 100)
T790M – 300 cp/mL	71	71	100 (94.9, 100)
L858R – 300 cp/mL	72	72	100 (95.0, 100)
L861Q – 300 cp/mL	71	71	100 (94.9, 100)

CI, confidence interval; cp, copies; Ex19Del, exon 19 deletion; Ex20Ins, exon 20 insertion; N/A, not applicable; OPA, overall percentage agreement.

### Interference substances

An interference study was performed to determine whether high levels of hemoglobin, triglycerides, bilirubin, or albumin, or the preservative solution in plasma from the Roche cfDNA tubes, would interfere or influence the performance of the cobas EGFR test.

All results gave the correct mutation call for all tested samples (Ex19Del, Ex20Ins, S768I, G719A, L861Q, T790M, L858R, and wildtype) for each condition. The tested potential interfering substances had no impact on the performance of the cobas EGFR test.

### Specimen stability

The stability and storage conditions of whole blood and plasma obtained from patients with NSCLC from the Roche cfDNA tubes were evaluated with the cobas EGFR test. Stability of NSCLC specimens was evaluated using five conditions, which included at least one wildtype and five *EGFR* mutation-positive samples. For each condition, one replicate per patient was tested at Time 0 and each time point: time (T0) (no storage of whole blood); Condition 1 (storage of whole blood for 25 hours at 32°C followed by storage at 27°C for 4 days [T1], 7 days [T2], and 8 days [T3]); Condition 2 (storage of plasma at 32°C for 5 hours [T4] and 25 hours [T5]); Condition 3 (storage of plasma at 2°C to 8°C for 4 days [T6] and 8 days [T7]; Condition 4 (storage of plasma at –25°C to –15°C for 31 days [T8] and 13 months [T9] with 3× freeze/thaw); and Condition 5 (storage of plasma at ≤–70°C for 31 days [T10] and 13 months [T11] with 3× freeze/thaw.

Results from the study were compared with T0 for each condition (Conditions 1 to 5; Table 8). Initially, Condition 1 was tested in comparison with T0. Once the whole blood storage condition was determined (T3), the subsequent testing of Conditions 2, 3, 4, and 5 followed Condition 1 T3 to separate the plasma prior to evaluating plasma storage. As a consequence, the NSCLC specimens used for each condition varied; however, the specimens tested for a given condition and its corresponding T0 were the same. *EGFR* wildtype and mutation-positive results from each patient at each time point had the same results from the corresponding T0 *EGFR* mutation-negative and mutation-positive samples. Results from the study further showed that whole blood collected in Roche cfDNA tubes can be stored for up to 25 hours at 32°C followed by up to 8 days at 27°C (Condition 1, T3), and following whole blood storage indicated above, plasma may be stored for up to: 1) 25 hours at 32°C (Condition 2, T5); 2) 8 days at 2°C to 8°C (Condition 3, T7); 3) 13 months at –25°C to –15°C (Condition 4, T9) with 3× freeze/thaw; and 4) 13 months at ≤–70°C (Condition 5, T11) with 3× freeze/thaw.

**Table 8:** Summary of specimen handling stability results.

Condition	Time point	Temperature	Time	Pass/Fail^a^
C1 (whole blood)	T1	32°C + 27°C	25 hours + 4 days	Pass
	T2	32°C + 27°C	25 hours + 7 days	Pass
	T3	32°C + 27°C	25 hours + 8 days	Pass
C2	T4	32°C	5 hours	Pass
	T5	32°C	25 hours	Pass
C3	T6	2°C to 8°C	4 days	Pass
	T7	2°C to 8°C	8 days	Pass
C4	T8	–25°C to –15°C	31 days	Pass
	T9	–25°C to –15°C	13 months	Pass
C5	T10	≤–70°C	31 days	Pass
	T11	≤–70°C	13 months	Pass

^a^A “Pass” result indicates no impact on the cobas EGFR test and that all results are valid with the correct mutation call.

## Discussion

The detection of ctDNA in liquid biopsy (plasma), has become increasingly used to detect clinically actionable mutations in patients with cancer. However, DNA has a short half-life in plasma and can be affected by the lysis of white blood cells present in blood, which increases the background DNA and thus dilutes the ctDNA present. Plasma must be separated from whole blood within 4 to 8 hours to prevent these challenges when blood is collected in standard K2EDTA blood collection tubes. In comparison, the Roche cfDNA tubes contain an anticoagulant (K3EDTA), as well as an inhibitor of cell lysis, which reduces the urgency for plasma separation, allowing laboratories greater flexibility in transportation and time to processing of blood for ctDNA detection. Using the Roche cfDNA tubes, blood specimens are stable for 7 days when stored or shipped between 15°C and 25°C, with transient excursions of up to 16 hours to 15°C to 30°C [24]. Previously, it was unknown whether the K3EDTA or the cell lysis inhibitor affected the performance of assays designed to detect mutations in ctDNA.

Using the cobas EGFR test, which is FDA-approved for detection of *EGFR* mutations in plasma collected in K2EDTA tubes from patients with NSCLC [25], this analysis evaluated the performance of the Roche cfDNA tube. Based on this analysis, surrogate samples were a reasonable substitute for NSCLC plasma based on LoD verification, surrogate sample study, and clinical LoD verification. This combination of studies demonstrated that surrogate sample performance directly translated to clinical sample verification.

This analysis also found that the performance of the cobas EGFR test was equally robust with either type of blood collection tube, eg, with regards to LoD, linearity, and reproducibility [25]. Further, the results for the linearity study also showed the utility of the SQI data for result interpretation, ie, the analysis showed a linear dose response over a wide range of input values. Moreover, the Roche cfDNA tube provides more flexibility for handling patient specimens than the K2EDTA tubes, eg, in terms of tube mixing, plasma separation, and sample stability, where this analysis included plasma samples stored long term for up to 13 months. In addition, the presence of potentially interfering substances did not impact the performance of the cobas EGFR test. As such, the Roche cfDNA tube can be used interchangeably with K2EDTA tubes when performing ctDNA testing for *EGFR* mutations with the cobas EGFR test.

The cobas EGFR test was previously shown to have robust accuracy and reproducibility in both preclinical and clinical studies using plasma collected in standard EDTA anticoagulation blood draw tubes. This study shows that the cobas EGFR test is similarly robust when using plasma obtained in Roche cfDNA tubes for up to 7 days, indicating that cfDNA remains detectable within this timeframe, thus making them suitable for routine clinical laboratory use in this setting. Furthermore, due to the preservative solution, the Roche cfDNA tubes enable draws that are not dependent upon processing within 8 hours, therefore increasing the reach of plasma biopsies for patients with NSCLC.

## Supporting information

S1 Fig Representative plasma image for mixing by inversion. ^a a^The number of mixes corresponds to the total number of inversions after cell-line DNA addition.
(DOCX)

S1 Table Comparison between surrogate samples and reference panel for Ex19Del (*n* = 20). ^a^0× LoD level for Panel 2 and 4 is shared; the same NSCLC wildtype plasma pool was used for both panels. cp, copies; CI, confidence interval; HD, healthy donor; Ex19Del, exon 19 deletion; LoD, limit of detection; N/A, not applicable; NSCLC, non-small cell lung cancer.
(DOCX)

S2 Table Comparison between surrogate samples and reference panel for L858R (*n* = 20). ^a^0× LoD level for Panel 2 and 4 is shared; the same NSCLC wildtype plasma pool was used for both panels. cp, copies; HD, healthy donor; LoD, limit of detection; N/A, not applicable; NSCLC, non-small cell lung cancer.
(DOCX)

S3 Table Comparison between surrogate samples and reference panel for T790M (*n* = 20). ^a^0× LoD level for Panel 2 and 4 is shared; the same NSCLC wildtype plasma pool was used for both panels. cp, copies; HD, healthy donor; LoD, limit of detection; N/A, not applicable; NSCLC, non-small cell lung cancer.
(DOCX)

S4 Table Inversions for mixing. ^a^A “Pass” result indicates no impact on the cobas EGFR test and that all results are valid with the correct mutation call. ^b^Control condition.
(DOCX)

S5 Table Centrifugation conditions. Samples used in the study included Ex19Del, EX20Ins, S768I, G719A, L861Q, T790M, L858R, and wildtype. ^a^A “Pass” result indicates no impact on the cobas EGFR test and that all results are valid with the correct mutation call. ^b^Control condition (center of the recommended range of 10 to 15 minutes of centrifugation time). Ex19Del, exon 19 deletion; Ex20Ins, exon 20 insertion.
(DOCX)

S6 Table Concentration range for each *EGFR* mutation group. cp, copies; Ex19Del, exon 19 deletion; Ex20Ins, exon 20 insertion.
(DOCX)

S7 Table Predicted SQI from regression analysis for Ex19Del. cp, copies; Ex19Del, exon 19 deletion; SD, standard deviation; SQI, Semi-Quantitative Index.
(DOCX)

S8 Table Predicted SQI from regression analysis for S768I. cp, copies; SD, standard deviation; SQI, Semi-Quantitative Index.
(DOCX)

S9 Table Predicted SQI from regression analysis for L858R. ^a^Six of eight replicates were positive for an *EGFR* mutation at this concentration, which is below the LoD. cp, copies; LoD, limit of detection; SD, standard deviation; SQI, Semi-Quantitative Index.
(DOCX)

S10 Table Predicted SQI from regression analysis for T790M. cp, copies; SD, standard deviation; SQI, Semi-Quantitative Index.
(DOCX)

S11 Table Predicted SQI from regression analysis for L861Q. ^a^Five of eight replicates were positive for an EGFR mutation at this concentration, which is below the LoD. cp, copies; LoD, limit of detection; SD, standard deviation; SQI, Semi-Quantitative Index.
(DOCX)

S12 Table Predicted SQI from regression analysis for G719X. cp, copies; N/A, not applicable; SD, standard deviation; SQI, Semi-Quantitative Index.
(DOCX)

S13 Table Predicted SQI from regression analysis for Ex20Ins. ^a^Two of eight replicates were positive for an *EGFR* mutation at this concentration, which is below the LoD. cp, copies; Ex20Ins, exon 20 insertion; LoD, limit of detection; SD, standard deviation; SQI, Semi-Quantitative Index.
(DOCX)

S14 Table Minimum SQI required for *EGFR* mutations. ^a^The SQI is a semi-quantitative measure of the amount of mutation-positive cfDNA in a sample that correlates with cp/mL and can be used to measure the presence of *EGFR* mutations from serial collections of plasma. An increase in the SQI value indicates an increase in the amount of the corresponding target mutation within an individual sample source, whereas a decrease in the SQI value indicates a decrease in the overall amount of the corresponding target mutation within an individual sample source. SQI is not available in the US as a diagnostic tool. cfDNA, cell-free DNA; cp, copies; Ex19Del, exon 19 deletion; Ex20Ins, exon 20 insertion; SQI, Semi-Quantitative Index.
(DOCX)

S15 Table Overall CV for the cycle threshold from valid mutation members. cp, copies; CV, coefficient of variation; Ex19Del, exon 19 deletion; Ex20Ins, exon 20 insertion.
(DOCX)
