# Supplementary material for: Performance characteristics of a polymerase chain reaction-based assay for the detection of EGFR mutations in plasma cell-free DNA from patients with non-small cell lung cancer using cell-free DNA collection tubes
Source: PLoS One. 2024 Apr 9;19(4):e0295987. doi: 10.1371/journal.pone.0295987 (PMC11003689; doi:10.1371/journal.pone.0295987)

**S1 Fig. Representative plasma image for mixing by inversion.^a^**

^a^The number of mixes corresponds to the total number of inversions after cell-line DNA addition.
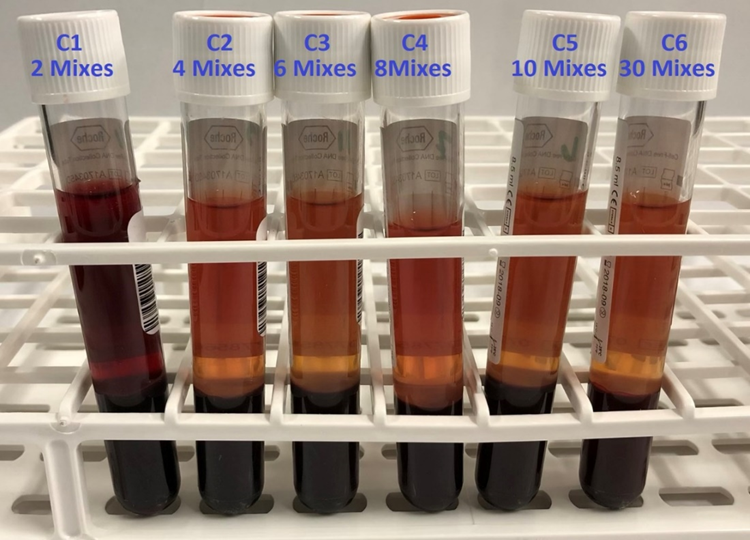

Supplement: S1 Fig — a aThe number of mixes corresponds to the total number of inversions after cell-line DNA addition. (DOCX) [file pone.0295987.s001.docx]
