# Supplementary material for: Performance characteristics of a polymerase chain reaction-based assay for the detection of EGFR mutations in plasma cell-free DNA from patients with non-small cell lung cancer using cell-free DNA collection tubes
Source: PLoS One. 2024 Apr 9;19(4):e0295987. doi: 10.1371/journal.pone.0295987 (PMC11003689; doi:10.1371/journal.pone.0295987)
Supplement: S2 Table — a0× LoD level for Panel 2 and 4 is shared; the same NSCLC wildtype plasma pool was used for both panels. cp, copies; HD, healthy donor; LoD, limit of detection; N/A, not applicable; NSCLC, non-small cell lung cancer. (DOCX) [file pone.0295987.s003.docx]

**S2 Table. Comparison between surrogate samples and reference panel for L858R (*n* = 20).**

|  | **Hit rate, % (95% CI)** | | | | | | |
| --- | --- | --- | --- | --- | --- | --- | --- |
| **Concentration× LoD (cp/mL)** | **Sheared cell-line DNA in HD plasma (Panel 1)** | **Sheared cell-line DNA in *EGFR* wildtype NSCLC plasma (Panel 2)** | | | **Plasma from HD whole blood spiked with sheared cell-line DNA (Panel 3)** | | ***EGFR* mutation-positive NSCLC plasma (Panel 4)  (Reference)** |
| 2× LoD (200 cp/mL) | 100 (83.2–100) | | 100 (83.2–100) | | | 100 (83.2–100) | 100 (83.2–100) |
| 1× LoD (100 cp/mL) | 100 (83.2–100) | | 100 (83.2–100) | | | 100 (83.2–100) | 100 (83.2–100) |
| 0.5× LoD (50 cp/mL) | 100 (83.2–100) | | 100 (83.2–100) | | | 100 (83.2–100) | 100 (83.2–100) |
| 0.13× LoD (13 cp/mL) | 100 (83.2–100) | | 100 (75.1–99.9) | | | 100 (83.2–100) | 85 (62.1–93.9) |
| 0.03× LoD (3 cp/mL) | 75.0 (50.9–91.3) | | 40 (19.1–63.9) | | | 60 (36.1–80.9) | 40 (19.1–63.9) |
| 0× LoD (0 cp/mL) | 0 (0–16.8) | | | 0^a^ (0–16.8) | | 0 (0.1–24.9) | 0^a^ (0–16.8) |
| ^a^0× LoD level for Panel 2 and 4 is shared; the same NSCLC wildtype plasma pool was used for both panels.  cp, copies; HD, healthy donor; LoD, limit of detection; N/A, not applicable; NSCLC, non-small cell lung cancer. | | | | | | | |
