# Supplementary material for: Performance characteristics of a polymerase chain reaction-based assay for the detection of EGFR mutations in plasma cell-free DNA from patients with non-small cell lung cancer using cell-free DNA collection tubes
Source: PLoS One. 2024 Apr 9;19(4):e0295987. doi: 10.1371/journal.pone.0295987 (PMC11003689; doi:10.1371/journal.pone.0295987)
Supplement: S4 Table — aA “Pass” result indicates no impact on the cobas EGFR test and that all results are valid with the correct mutation call. bControl condition. (DOCX) [file pone.0295987.s005.docx]

**S4 Table. Inversions for mixing.**

| **Condition** | **Number of inversions after blood draw** | **Total number of inversions after cell-line DNA addition** | **Result (Pass or Fail)^a^** |
| --- | --- | --- | --- |
| C1 | 0 | 2 | Pass |
| C2 | 2 | 4 | Pass |
| C3 | 4 | 6 | Pass |
| **C4^b^** | **6** | **8** | Pass |
| C5 | 8 | 10 | Pass |
| C6 | 28 | 30 | Pass |

^a^A “Pass” result indicates no impact on the cobas EGFR test and that all results are valid with the correct mutation call.

^b^Control condition.
