# Supplementary material for: Performance characteristics of a polymerase chain reaction-based assay for the detection of EGFR mutations in plasma cell-free DNA from patients with non-small cell lung cancer using cell-free DNA collection tubes
Source: PLoS One. 2024 Apr 9;19(4):e0295987. doi: 10.1371/journal.pone.0295987 (PMC11003689; doi:10.1371/journal.pone.0295987)
Supplement: S5 Table — Samples used in the study included Ex19Del, EX20Ins, S768I, G719A, L861Q, T790M, L858R, and wildtype. aA “Pass” result indicates no impact on the cobas EGFR test and that all results are valid with the correct mutation call. bControl condition (center of the recommended range of 10 to 15 minutes of centrifugation time). Ex19Del, exon 19 deletion; Ex20Ins, exon 20 insertion. (DOCX) [file pone.0295987.s006.docx]

**S5 Table. Centrifugation conditions.**

| **Condition** | **Centrifugation speed (×g)** | **Centrifugation time (minutes)** | **Result (Pass or Fail)^a^** |
| --- | --- | --- | --- |
| C1 | 2600 | 9 | Pass |
| C2 | 2600 | 16 | Pass |
| C3**^b^** | 1600 | 12.5 | Pass |
| C4 | 1100 | 9 | Pass |
| C5 | 1100 | 16 | Pass |

Samples used in the study included Ex19Del, EX20Ins, S768I, G719A, L861Q, T790M, L858R, and wildtype.

^a^A “Pass” result indicates no impact on the cobas EGFR test and that all results are valid with the correct mutation call.

^b^Control condition (center of the recommended range of 10 to 15 minutes of centrifugation time).

Ex19Del, exon 19 deletion; Ex20Ins, exon 20 insertion.
