# Supplementary material for: Performance characteristics of a polymerase chain reaction-based assay for the detection of EGFR mutations in plasma cell-free DNA from patients with non-small cell lung cancer using cell-free DNA collection tubes
Source: PLoS One. 2024 Apr 9;19(4):e0295987. doi: 10.1371/journal.pone.0295987 (PMC11003689; doi:10.1371/journal.pone.0295987)
Supplement: S6 Table — cp, copies; Ex19Del, exon 19 deletion; Ex20Ins, exon 20 insertion. (DOCX) [file pone.0295987.s007.docx]

**S6 Table.** **Concentration range for each *EGFR* mutation group.**

| ***EGFR* mutation group** | **Concentration range** | |
| --- | --- | --- |
|  | **(cp/mL)** | **(log_10_ cp/mL)** |
| Ex19Del | 1.0x10^1^ to 1.0x10^5^ | 1.0 to 5.0 |
| S768I | 1.0x10^1^ to 1.0x10^5^ | 1.0 to 5.0 |
| L858R | 1.0x10^1^ to 1.0x10^5^ | 1.0 to 5.0 |
| T790M | 5.0x10^1^ to 1.0x10^5^ | 1.7 to 5.0 |
| L861Q | 1.0x10^1^ to 1.0x10^5^ | 1.0 to 5.0 |
| G719X | 5.0x10^1^ to 1.0x10^4^ | 1.7 to 4.0 |
| Ex20Ins | 1.0x10^1^ to 1.0x10^5^ | 1.0 to 5.0 |

cp, copies; Ex19Del, exon 19 deletion; Ex20Ins, exon 20 insertion.
