# Supplementary material for: Performance characteristics of a polymerase chain reaction-based assay for the detection of EGFR mutations in plasma cell-free DNA from patients with non-small cell lung cancer using cell-free DNA collection tubes
Source: PLoS One. 2024 Apr 9;19(4):e0295987. doi: 10.1371/journal.pone.0295987 (PMC11003689; doi:10.1371/journal.pone.0295987)
Supplement: S7 Table — cp, copies; Ex19Del, exon 19 deletion; SD, standard deviation; SQI, Semi-Quantitative Index. (DOCX) [file pone.0295987.s008.docx]

**S7 Table.** **Predicted SQI from regression analysis for Ex19Del.**

| ***EGFR* mutation group** | **Panel member** | **Concentration (cp/mL)** | **Log (cp/mL)** | ***N*** | **Average SQI** | **SQI SD** | **Predicted SQI based on regression analysis** | | | **Difference from linear fit** |
| --- | --- | --- | --- | --- | --- | --- | --- | --- | --- | --- |
|  |  |  |  |  |  |  | **First order [linear]** | **Second order** | **Third order** | **Third–first order** |
| Ex19Del | 1 | 1.0 × 10^5^ | 5.0 | 4 | 24.30 | 0.19 | 24.22 | 24.23 | 24.27 | 0.05 |
|  | 2 | 1.0 × 10^4^ | 4.0 | 8 | 20.86 | 0.20 | 20.95 | 20.95 | 20.92 | –0.03 |
|  | 3 | 3.2 × 10^3^ | 3.5 | 8 | 19.31 | 0.22 | 19.31 | 19.31 | 19.29 | –0.02 |
|  | 4 | 1.0 × 10^3^ | 3.0 | 8 | 17.70 | 0.17 | 17.67 | 17.67 | 17.67 | 0.00 |
|  | 5 | 3.2 × 10^2^ | 2.5 | 8 | 16.11 | 0.11 | 16.03 | 16.03 | 16.05 | 0.02 |
|  | 6 | 1.0 × 10^2^ | 2.0 | 8 | 14.34 | 0.29 | 14.40 | 14.40 | 14.43 | 0.03 |
|  | 7 | 1.0 × 10^1^ | 1.0 | 8 | 11.12 | 0.51 | 11.12 | 11.12 | 11.10 | –0.02 |

cp, copies; Ex19Del, exon 19 deletion; SD, standard deviation; SQI, Semi-Quantitative Index.
