# Supplementary material for: Performance characteristics of a polymerase chain reaction-based assay for the detection of EGFR mutations in plasma cell-free DNA from patients with non-small cell lung cancer using cell-free DNA collection tubes
Source: PLoS One. 2024 Apr 9;19(4):e0295987. doi: 10.1371/journal.pone.0295987 (PMC11003689; doi:10.1371/journal.pone.0295987)
Supplement: S8 Table — cp, copies; SD, standard deviation; SQI, Semi-Quantitative Index. (DOCX) [file pone.0295987.s009.docx]

**S8 Table.** **Predicted SQI from regression analysis for S768I.**

| ***EGFR* mutation group** | **Panel member** | **Concentration (cp/mL)** | **Log (cp/mL)** | ***N*** | **Average SQI** | **SQI SD** | **Predicted SQI based on regression analysis** | | | **Difference from linear fit** |
| --- | --- | --- | --- | --- | --- | --- | --- | --- | --- | --- |
|  |  |  |  |  |  |  | **First order [linear]** | **Second order** | **Third order** | **Third–First** |
| S768I | 1 | 1.0 × 10^5^ | 5.0 | 4 | 16.10 | 0.10 | 15.57 | 16.15 | 16.10 | 0.53 |
|  | 2 | 1.0 × 10^4^ | 4.0 | 8 | 12.52 | 0.14 | 12.43 | 12.44 | 12.47 | 0.04 |
|  | 3 | 3.2 × 10^3^ | 3.5 | 8 | 10.66 | 0.29 | 10.86 | 10.72 | 10.74 | –0.12 |
|  | 4 | 1.0 × 10^3^ | 3.0 | 8 | 9.09 | 0.24 | 9.30 | 9.08 | 9.08 | –0.22 |
|  | 5 | 3.2 × 10^2^ | 2.5 | 8 | 7.59 | 0.26 | 7.73 | 7.53 | 7.51 | –0.22 |
|  | 6 | 1.0 × 10^2^ | 2.0 | 8 | 5.98 | 0.44 | 6.16 | 6.06 | 6.03 | –0.13 |
|  | 7 | 1.0 × 10^1^ | 1.0 | 8 | 3.41 | 0.35 | 3.03 | 3.39 | 3.41 | 0.38 |

cp, copies; SD, standard deviation; SQI, Semi-Quantitative Index.
