# Supplementary material for: Performance characteristics of a polymerase chain reaction-based assay for the detection of EGFR mutations in plasma cell-free DNA from patients with non-small cell lung cancer using cell-free DNA collection tubes
Source: PLoS One. 2024 Apr 9;19(4):e0295987. doi: 10.1371/journal.pone.0295987 (PMC11003689; doi:10.1371/journal.pone.0295987)
Supplement: S9 Table — aSix of eight replicates were positive for an EGFR mutation at this concentration, which is below the LoD. cp, copies; LoD, limit of detection; SD, standard deviation; SQI, Semi-Quantitative Index. (DOCX) [file pone.0295987.s010.docx]

**S9 Table.** **Predicted SQI from regression analysis for L858R.**

| ***EGFR* mutation group** | **Panel member** | **Concentration (cp/mL)** | **Log (cp/mL)** | ***N*** | **Average SQI** | **SQI SD** | **Predicted SQI based on regression analysis** | | | **Difference from linear fit** |
| --- | --- | --- | --- | --- | --- | --- | --- | --- | --- | --- |
|  |  |  |  |  |  |  | **First order [linear]** | **Second order** | **Third order** | **Third–First** |
| L858R | 1 | 1.0 × 10^5^ | 5.0 | 4 | 18.44 | 0.09 | 18.95 | 18.67 | 18.45 | –0.28 |
|  | 2 | 1.0 × 10^4^ | 4.0 | 8 | 16.03 | 0.15 | 15.85 | 15.85 | 16.02 | 0.00 |
|  | 3 | 3.2 × 10^3^ | 3.5 | 8 | 14.48 | 0.16 | 14.31 | 14.38 | 14.50 | 0.07 |
|  | 4 | 1.0 × 10^3^ | 3.0 | 8 | 12.89 | 0.15 | 12.76 | 12.86 | 12.87 | 0.10 |
|  | 5 | 3.2 × 10^2^ | 2.5 | 8 | 11.15 | 0.23 | 11.21 | 11.30 | 11.19 | 0.09 |
|  | 6 | 1.0 × 10^2^ | 2.0 | 8 | 9.55 | 0.17 | 9.66 | 9.70 | 9.52 | 0.04 |
|  | 7 | 1.0 × 10^1^ | 1.0 | 6^a^ | 6.50 | 0.51 | 6.57 | 6.36 | 6.51 | –0.21 |

^a^Six of eight replicates were positive for an *EGFR* mutation at this concentration, which is below the LoD.

cp, copies; LoD, limit of detection; SD, standard deviation; SQI, Semi-Quantitative Index.
