# Supplementary material for: Performance characteristics of a polymerase chain reaction-based assay for the detection of EGFR mutations in plasma cell-free DNA from patients with non-small cell lung cancer using cell-free DNA collection tubes
Source: PLoS One. 2024 Apr 9;19(4):e0295987. doi: 10.1371/journal.pone.0295987 (PMC11003689; doi:10.1371/journal.pone.0295987)
Supplement: S10 Table — cp, copies; SD, standard deviation; SQI, Semi-Quantitative Index. (DOCX) [file pone.0295987.s011.docx]

**S10 Table.** **Predicted SQI from regression analysis for T790M.**

| ***EGFR* mutation group** | **Panel member** | **Concentration (cp/mL)** | **Log (cp/mL)** | ***N*** | **Average SQI** | **SQI SD** | **Predicted SQI based on regression analysis** | | | **Difference from linear fit** |
| --- | --- | --- | --- | --- | --- | --- | --- | --- | --- | --- |
|  |  |  |  |  |  |  | **First order [linear]** | **Second order** | **Third order** | **Third–First** |
| T790M | 1 | 1.0 × 10^5^ | 5.0 | 4 | 21.35 | 0.21 | 21.16 | 21.27 | 21.35 | 0.11 |
|  | 2 | 1.0 × 10^4^ | 4.0 | 8 | 17.54 | 0.40 | 17.63 | 17.62 | 17.54 | –0.01 |
|  | 3 | 3.2 × 10^3^ | 3.5 | 8 | 15.80 | 0.33 | 15.87 | 15.82 | 15.79 | –0.05 |
|  | 4 | 1.0 × 10^3^ | 3.0 | 8 | 14.09 | 0.27 | 14.10 | 14.06 | 14.09 | –0.04 |
|  | 5 | 3.2 × 10^2^ | 2.5 | 8 | 12.38 | 0.26 | 12.33 | 12.31 | 12.38 | –0.02 |
|  | 6 | 1.0 × 10^2^ | 2.0 | 8 | 10.61 | 0.40 | 10.57 | 10.59 | 10.61 | 0.02 |
|  | 7 | 1.0 × 10^1^ | 1.7 | 8 | 9.50 | 0.47 | 9.51 | 9.56 | 9.50 | 0.05 |

cp, copies; SD, standard deviation; SQI, Semi-Quantitative Index.
