# Supplementary material for: Performance characteristics of a polymerase chain reaction-based assay for the detection of EGFR mutations in plasma cell-free DNA from patients with non-small cell lung cancer using cell-free DNA collection tubes
Source: PLoS One. 2024 Apr 9;19(4):e0295987. doi: 10.1371/journal.pone.0295987 (PMC11003689; doi:10.1371/journal.pone.0295987)
Supplement: S11 Table — aFive of eight replicates were positive for an EGFR mutation at this concentration, which is below the LoD. cp, copies; LoD, limit of detection; SD, standard deviation; SQI, Semi-Quantitative Index. (DOCX) [file pone.0295987.s012.docx]

**S11 Table.** **Predicted SQI from regression analysis for L861Q.**

| ***EGFR* mutation group** | **Panel member** | **Concentration (cp/mL)** | **Log (cp/mL)** | ***N*** | **Average SQI** | **SQI SD** | **Predicted SQI based on regression analysis** | | | **Difference from linear fit** |
| --- | --- | --- | --- | --- | --- | --- | --- | --- | --- | --- |
|  |  |  |  |  |  |  | **First order [linear]** | **Second order** | **Third order** | **Third–First** |
| L861Q | 1 | 1.0 × 10^5^ | 5.0 | 4 | 15.24 | 0.19 | 14.91 | 15.31 | 15.22 | 0.40 |
|  | 2 | 1.0 × 10^4^ | 4.0 | 8 | 11.69 | 0.14 | 11.70 | 11.69 | 11.76 | –0.01 |
|  | 3 | 3.2 × 10^3^ | 3.5 | 8 | 10.13 | 0.18 | 10.09 | 9.98 | 10.03 | –0.11 |
|  | 4 | 1.0 × 10^3^ | 3.0 | 8 | 8.30 | 0.16 | 8.49 | 8.34 | 8.34 | –0.15 |
|  | 5 | 3.2 × 10^2^ | 2.5 | 8 | 6.73 | 0.20 | 6.88 | 6.75 | 6.70 | –0.13 |
|  | 6 | 1.0 × 10^2^ | 2.0 | 8 | 5.14 | 0.28 | 5.27 | 5.24 | 5.17 | –0.03 |
|  | 7 | 1.0 × 10^1^ | 1.0 | 5^a^ | 2.50 | 0.33 | 2.06 | 2.41 | 2.49 | 0.35 |

^a^Five of eight replicates were positive for an *EGFR* mutation at this concentration, which is below the LoD.

cp, copies; LoD, limit of detection; SD, standard deviation; SQI, Semi-Quantitative Index.
