# Supplementary material for: Performance characteristics of a polymerase chain reaction-based assay for the detection of EGFR mutations in plasma cell-free DNA from patients with non-small cell lung cancer using cell-free DNA collection tubes
Source: PLoS One. 2024 Apr 9;19(4):e0295987. doi: 10.1371/journal.pone.0295987 (PMC11003689; doi:10.1371/journal.pone.0295987)
Supplement: S12 Table — cp, copies; N/A, not applicable; SD, standard deviation; SQI, Semi-Quantitative Index. (DOCX) [file pone.0295987.s013.docx]

**S12 Table.** **Predicted SQI from regression analysis for G719X.**

| ***EGFR* mutation group** | **Panel member** | **Concentration (cp/mL)** | **Log (cp/mL)** | ***N*** | **Average SQI** | **SQI SD** | **Predicted SQI based on regression analysis** | | | **Difference from linear fit** |
| --- | --- | --- | --- | --- | --- | --- | --- | --- | --- | --- |
|  |  |  |  |  |  |  | **First order [linear]** | **Second order** | **Third order** | **Third–First** |
| G719X | 1 | 1.0 × 10^5^ | 5.0 | N/A | N/A | N/A | N/A | N/A | N/A | N/A |
|  | 2 | 1.0 × 10^4^ | 4.0 | 8 | 10.39 | 0.09 | 10.39 | 10.39 | 10.40 | 0.01 |
|  | 3 | 3.2 × 10^3^ | 3.5 | 8 | 8.84 | 0.16 | 8.83 | 8.83 | 8.82 | –0.01 |
|  | 4 | 1.0 × 10^3^ | 3.0 | 8 | 7.24 | 0.19 | 7.27 | 7.27 | 7.27 | 0.00 |
|  | 5 | 3.2 × 10^2^ | 2.5 | 8 | 5.73 | 0.23 | 5.71 | 5.71 | 5.72 | 0.01 |
|  | 6 | 1.0 × 10^2^ | 2.0 | 8 | 4.17 | 0.21 | 4.15 | 4.15 | 4.16 | 0.01 |
|  | 7 | 1.0 × 10^1^ | 1.7 | 8 | 3.20 | 0.27 | 3.21 | 3.21 | 3.20 | –0.01 |

cp, copies; N/A, not applicable; SD, standard deviation; SQI, Semi-Quantitative Index.
