# Supplementary material for: Performance characteristics of a polymerase chain reaction-based assay for the detection of EGFR mutations in plasma cell-free DNA from patients with non-small cell lung cancer using cell-free DNA collection tubes
Source: PLoS One. 2024 Apr 9;19(4):e0295987. doi: 10.1371/journal.pone.0295987 (PMC11003689; doi:10.1371/journal.pone.0295987)
Supplement: S13 Table — aTwo of eight replicates were positive for an EGFR mutation at this concentration, which is below the LoD. cp, copies; Ex20Ins, exon 20 insertion; LoD, limit of detection; SD, standard deviation; SQI, Semi-Quantitative Index. (DOCX) [file pone.0295987.s014.docx]

**S13 Table.** **Predicted SQI from regression analysis for Ex20Ins.**

| ***EGFR* mutation group** | **Panel member** | **Concentration (cp/mL)** | **Log (cp/mL)** | ***N*** | **Average SQI** | **SQI SD** | **Predicted SQI based on regression analysis** | | | **Difference from linear fit** |
| --- | --- | --- | --- | --- | --- | --- | --- | --- | --- | --- |
|  |  |  |  |  |  |  | **First order [linear]** | **Second order** | **Third order** | **Third–First** |
| Ex20Ins | 1 | 1.0 × 10^5^ | 5.0 | 4 | 14.57 | 0.08 | 14.11 | 14.58 | 14.62 | 0.51 |
|  | 2 | 1.0 × 10^4^ | 4.0 | 8 | 10.81 | 0.10 | 10.81 | 10.77 | 10.74 | –0.07 |
|  | 3 | 3.2 × 10^3^ | 3.5 | 8 | 9.04 | 0.20 | 9.16 | 9.00 | 8.98 | –0.18 |
|  | 4 | 1.0 × 10^3^ | 3.0 | 8 | 7.20 | 0.14 | 7.51 | 7.33 | 7.33 | –0.18 |
|  | 5 | 3.2 × 10^2^ | 2.5 | 8 | 5.65 | 0.16 | 5.86 | 5.76 | 5.78 | –0.08 |
|  | 6 | 1.0 × 10^2^ | 2.0 | 8 | 4.49 | 0.26 | 4.21 | 4.28 | 4.30 | 0.09 |
|  | 7 | 1.0 × 10^1^ | 1.0 | 2^a^ | 1.39 | 0.16 | 0.91 | 1.60 | 1.53 | 0.62 |

^a^Two of eight replicates were positive for an *EGFR* mutation at this concentration, which is below the LoD.

cp, copies; Ex20Ins, exon 20 insertion; LoD, limit of detection; SD, standard deviation; SQI, Semi-Quantitative Index.
