# Supplementary material for: Performance characteristics of a polymerase chain reaction-based assay for the detection of EGFR mutations in plasma cell-free DNA from patients with non-small cell lung cancer using cell-free DNA collection tubes
Source: PLoS One. 2024 Apr 9;19(4):e0295987. doi: 10.1371/journal.pone.0295987 (PMC11003689; doi:10.1371/journal.pone.0295987)
Supplement: S14 Table — aThe SQI is a semi-quantitative measure of the amount of mutation-positive cfDNA in a sample that correlates with cp/mL and can be used to measure the presence of EGFR mutations from serial collections of plasma. An increase in the SQI value indicates an increase in the amount of the corresponding target mutation within an individual sample source, whereas a decrease in the SQI value indicates a decrease in the overall amount of the corresponding target mutation within an individual sample source. SQI is not available in the US as a diagnostic tool. cfDNA, cell-free DNA; cp, copies; Ex19Del, exon 19 deletion; Ex20Ins, exon 20 insertion; SQI, Semi-Quantitative Index. (DOCX) [file pone.0295987.s015.docx]

**S14 Table.** **Minimum SQI required for *EGFR* mutations.**

| ***EGFR* mutation group** | **Minimum SQI required^a^** |
| --- | --- |
| Ex19Del | 13.00 |
| S768I | 4.00 |
| L858R | 9.50 |
| T790M | 9.50 |
| L861Q | 3.00 |
| G719X | 4.00 |
| Ex20Ins | 2.50 |

^a^The SQI is a semi-quantitative measure of the amount of mutation-positive cfDNA in a sample that correlates with cp/mL and can be used to measure the presence of *EGFR* mutations from serial collections of plasma. An increase in the SQI value indicates an increase in the amount of the corresponding target mutation within an individual sample source, whereas a decrease in the SQI value indicates a decrease in the overall amount of the corresponding target mutation within an individual sample source. SQI is not available in the US as a diagnostic tool.

cfDNA, cell-free DNA; cp, copies; Ex19Del, exon 19 deletion; Ex20Ins, exon 20 insertion; SQI, Semi-Quantitative Index.
