# Supplementary material for: Performance characteristics of a polymerase chain reaction-based assay for the detection of EGFR mutations in plasma cell-free DNA from patients with non-small cell lung cancer using cell-free DNA collection tubes
Source: PLoS One. 2024 Apr 9;19(4):e0295987. doi: 10.1371/journal.pone.0295987 (PMC11003689; doi:10.1371/journal.pone.0295987)
Supplement: S15 Table — cp, copies; CV, coefficient of variation; Ex19Del, exon 19 deletion; Ex20Ins, exon 20 insertion. (DOCX) [file pone.0295987.s016.docx]

**S15 Table. Overall CV for the cycle threshold from valid mutation members.**

|  | **CV (%)** | | | | | | |
| --- | --- | --- | --- | --- | --- | --- | --- |
| **Panel member – level** | ***N*** | **Lot** | **Site** | **Operator** | **Day** | **Within–run** | **Total** |
| Ex19Del – 100 cp/mL | 72 | 3.6 | 2.9 | 0.5 | 2.0 | 3.3 | 6.0 |
| S768I – 100 cp/mL | 72 | 3.6 | 4.1 | 0.0 | 0.0 | 4.2 | 6.9 |
| L858R – 100 cp/mL | 72 | 6.6 | 2.6 | 0.0 | 3.8 | 7.3 | 10.9 |
| T790M – 100 cp/mL | 72 | 7.0 | 5.1 | 0.0 | 2.2 | 10.4 | 13.7 |
| L861Q – 100 cp/mL | 72 | 2.2 | 2.8 | 1.5 | 0.0 | 4.1 | 5.6 |
| G719A – 100 cp/mL | 72 | 3.5 | 1.8 | 0.8 | 0.0 | 4.8 | 6.3 |
| Ex20Ins – 100 cp/mL | 72 | 6.3 | 6.2 | 1.7 | 3.4 | 6.8 | 11.8 |
| Ex19Del – 300 cp/mL | 71 | 3.1 | 1.5 | 1.4 | 2.5 | 3.3 | 5.6 |
| S768I – 300 cp/mL | 72 | 4.7 | 3.2 | 0.8 | 0.9 | 3.1 | 6.5 |
| L858R – 300 cp/mL | 72 | 6.9 | 3.8 | 0.0 | 3.3 | 8.3 | 11.9 |
| T790M – 300 cp/mL | 71 | 5.1 | 2.4 | 1.4 | 0.0 | 8.1 | 10.0 |
| L861Q – 300 cp/mL | 71 | 2.3 | 3.2 | 0.7 | 2.3 | 3.4 | 5.7 |
| G719A – 300 cp/mL | 72 | 2.4 | 2.1 | 0.4 | 1.4 | 2.6 | 4.4 |
| Ex20Ins – 300 cp/mL | 71 | 6.1 | 7.9 | 0.0 | 2.9 | 7.0 | 12.5 |

cp, copies; CV, coefficient of variation; Ex19Del, exon 19 deletion; Ex20Ins, exon 20 insertion.
